# Unusual Presentation of Obstructive Atherosclerotic Coronary Artery Disease With Chronic, Persistent Neck and Shoulder Pain: A Case Report

**DOI:** 10.7759/cureus.80298

**Published:** 2025-03-09

**Authors:** Pacelli C Osigwe, Ifunanya S Osigwe, Amando A Obieze, Ebubechi Osigwe, Chukwudike E Agomoh

**Affiliations:** 1 Department of Cardiology, Bronglais General Hospital, Aberystwyth, GBR; 2 Department of Medicine, Bronglais General Hospital, Aberystwyth, GBR; 3 Department of Medicine, Worcestershire Royal Hospital, Worcester, GBR; 4 Department of General Practice, Improving Health (IH) Medical, Wolverhampton, GBR; 5 Department of General Practice, Hall Street Medical Centre, St. Helens, GBR

**Keywords:** atherosclerotic, case report, coronary artery disease, persistent neck pain, persistent shoulder pain

## Abstract

Ischaemic chest pain or its equivalents are acute-onset in acute coronary syndrome (ACS) and chronic, episodic, and transient in chronic coronary syndrome (CCS). A 56-year-old Caucasian male with a history of premature atherosclerotic coronary artery disease (CAD) presented to secondary care with recurrent presyncope and syncope. He reported a year-long history of persistent left-sided neck and shoulder dull ache/tightness, unrelated to exertion and fluctuating unpredictably. His primary care had diagnosed the pain as musculoskeletal, attributing it to prior physical trauma. However, the pain did not respond to treatment. During his admission for suspected cardiac syncope, he experienced transient chest discomfort, transient inferior ST-segment elevation on electrocardiogram (ECG), and elevated troponin levels, indicating a non-ST-elevation myocardial infarction (NSTEMI). Coronary angiography revealed obstructive atherosclerotic two-vessel disease, with severe proximal stenosis in the right coronary artery (RCA) and moderate-to-severe stenosis in the left anterior descending artery (LAD). His chronic neck and shoulder pain resolved after percutaneous coronary intervention (PCI) with drug-eluting stent (DES) placement in the RCA, confirming it was an anginal equivalent. Although the chronicity of this anginal equivalent may align it more with CCS than ACS, its unremitting nature is inconsistent with CCS. Our patient's history also showed that his ischaemic symptoms changed over time, from remote exertional dyspnoea to persistent neck and shoulder pain, and then to the chest discomfort that preceded his NSTEMI. Our case highlights the importance of heightened clinician awareness of atypical CAD presentations and symptom variability over time. Symptoms initially considered non-anginal should be reassessed for CAD, particularly when alternative treatments prove ineffective. Similar cases like ours, in the future, could prompt updates to CCS diagnostic guidelines to address atypical presentations with persistent pain.

## Introduction

Ischaemic heart disease (IHD) is a condition characterised by myocardial ischaemia, which occurs when there is a mismatch between the heart’s oxygen demand and its blood supply. IHD is commonly caused by atherosclerotic coronary artery disease (CAD) but can also result from other coronary pathologies, such as vasomotor abnormalities, microvascular dysfunction, congenital abnormalities, aneurysm, dissection, embolism, or intramural haematoma [1,2]. Additionally, non-coronary pathologies, such as anaemia, hypoxaemia, hypotension, hypertension, arrhythmias, myocardial hypertrophy, or myocardial fibrosis, can lead to myocardial ischaemia [1,2]. Cardiovascular disease (CVD), which encompasses a variety of diseases affecting the heart and/or blood vessels, is the leading cause of morbidity and mortality worldwide, with mortality rates mainly driven by IHD [3,4].

The principal symptom of myocardial ischaemia is angina, which is typically characterised as chest pain or discomfort. Angina is usually retrosternal and may be described as ‘heaviness,’ ‘pressure,’ ‘squeezing,’ ‘strangling,’ ‘tightness,’ ‘constricting,’ or ‘burning’ [1,2]. About 20%-30% of IHD patients do not experience angina but report anginal equivalents, such as jaw pain, neck pain, shoulder pain, arm pain, back pain, epigastric pain, indigestion, dyspnoea, palpitation, dizziness, syncope, diaphoresis, nausea/vomiting, or fatigue [1,2,5,6].

Chronic coronary syndromes (CCS) and acute coronary syndromes (ACS) encompass the various clinical presentations of myocardial ischaemia. CCS comprises the clinical manifestations of chronic coronary pathology, typically caused by atherosclerosis, vasomotor abnormalities, and/or microvascular dysfunction [1]. It may be asymptomatic or present with transient angina (or equivalent) due to reversible myocardial ischaemia [1]. Therefore, the chronic stable period of atherosclerotic CAD may present with transient (<10 minutes), predictable, reproducible stress-induced angina (or anginal equivalent) or may be asymptomatic and detected during other diagnostic workups [1].

Coronary atherosclerotic plaques can sustain acute events such as rupture, ulceration, fissure, or erosion [2]. These events may lead to territorial myocardial ischaemia due to superimposed intraluminal thrombosis and/or distal embolisation [2]. This acute destabilised phase of atherosclerotic CAD may present as one of the ACS: unstable angina (UA), non-ST-elevation myocardial infarction (NSTEMI), or ST-elevation myocardial infarction (STEMI). Clinically, ACS may be recognised by worsening characteristics of previously stable angina or anginal equivalent (increasing frequency, increasing duration, decreasing threshold, or occurrence at rest), new-onset severe angina (or anginal equivalent), or angina (or anginal equivalent) at rest persisting longer than 20 minutes [2]. STEMI is associated with persistent ST-segment elevation in contiguous electrocardiogram (ECG) leads and a dynamic rise in cardiac troponin [2]. UA and NSTEMI are not associated with persistent ST-segment elevation on ECG; however, there is a dynamic rise in troponin levels in NSTEMI but not in UA [2]. Myocardial infarction may also be asymptomatic or unrecognised, only detected following an ECG or cardiac imaging [7].

The degree of stenosis in epicardial coronary arteries caused by atherosclerotic plaques can be assessed using invasive coronary angiography. Obstructive atherosclerotic CAD refers to a reduction of at least 50% in the luminal diameter of a coronary artery [2]. In the right coronary artery (RCA), left anterior descending artery (LAD), or left circumflex artery (LCx), a 50%-69% reduction in the luminal diameter is regarded as moderate stenosis [1], while a ≥70% diameter reduction represents severe stenosis [8]. The threshold is different in the left main coronary artery (LMCA), where a diameter reduction of ≥50% is classified as severe stenosis [9,10].

We describe a 56-year-old man with unusual symptomatology of obstructive atherosclerotic CAD.

## Case presentation

A 56-year-old Caucasian male was brought to our secondary care hospital after three witnessed syncopal episodes within an hour. He reported three presyncopal episodes in the preceding six months. These presyncopal/syncopal episodes were not preceded by postural change, chest pain, palpitations, or exertion. He also reported a one-year history of ongoing, persistent, non-radiating left-sided neck and shoulder dull ache/tightness, which waxed and waned unpredictably. His pain started shortly after a road traffic collision a year before. He scored his pain as 3/10 or 4/10 on 'good days' and 10/10 on 'bad days.' On 'bad days,' pain disrupted his sleep. Waxing pain (10/10) always accompanied his presyncopal symptoms of dizziness, nausea, visual changes, and clamminess. He was physically active, mountain biking twice weekly, averaging 50 miles. He denied any exacerbation of his neck and shoulder pain (or any chest pain) during physical activity.

Evaluation of his pain at his primary healthcare included a normal cervical radiograph, and his pain was thought to be musculoskeletal, related to his road traffic collision. He was managed at his primary healthcare with analgesia (codeine-paracetamol) and physiotherapy, but he awaited a chiropractor appointment due to poor response to treatment.

His significant past medical history included hypertension, dyslipidaemia, and premature atherosclerotic CAD. His CAD was diagnosed 14 years earlier following a workup for dyspnoea on exertion. He underwent a percutaneous coronary intervention (PCI) with drug-eluting stent (DES) implantation in his LAD, with no recurrent symptoms afterward.

He had no significant family history of CAD. His routine medications included ramipril, low-dose aspirin, low-dose rosuvastatin, a proton pump inhibitor, and an antacid. He had a history of intolerance to simvastatin, atorvastatin, and ezetimibe, but no known drug allergies. He was an ex-smoker and consumed 10-20 units of alcohol weekly. A review of his systems was unremarkable.

His observations were remarkable for elevated blood pressure (150/105 mmHg) and bradycardia (52 beats per minute). His body mass index was 26.5 kg/m². General physical and systemic examinations were unremarkable.

**Figure 1:**
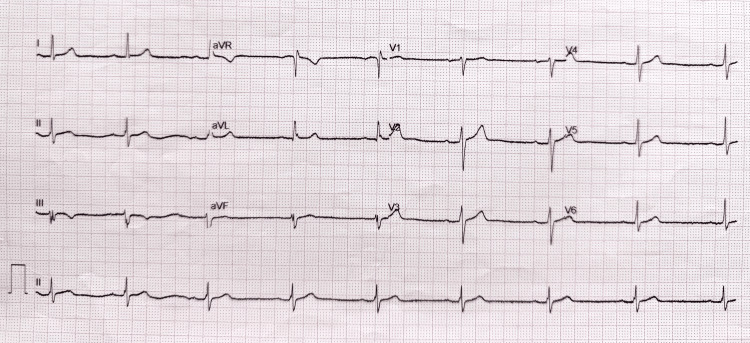
Electrocardiogram at hospital admission. An electrocardiogram recorded at admission showing sinus bradycardia at 50 beats per minute (bpm) and a borderline first-degree atrioventricular (AV) block.

Blood tests revealed normal cell counts, electrolytes, troponin, and renal and liver function. His ECG showed sinus bradycardia, borderline first-degree atrioventricular (AV) block, but no acute ischaemic changes (Figure 1). His chest radiograph was normal.

**Figure 2:**
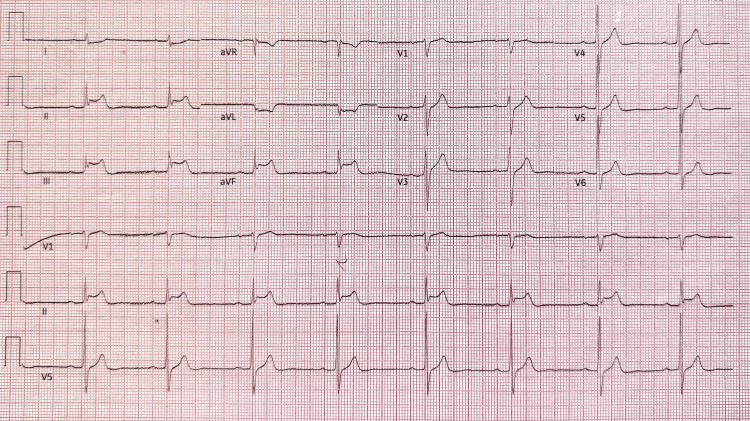
Initial electrocardiogram after retrosternal chest discomfort, showing ST-segment elevation in the inferior leads (II, III, and aVF). This figure also shows sinus bradycardia at 50 beats per minute (bpm), a borderline first-degree atrioventricular (AV) block, and reciprocal ST-segment depression in the lateral leads (I and aVL).

**Figure 3:**
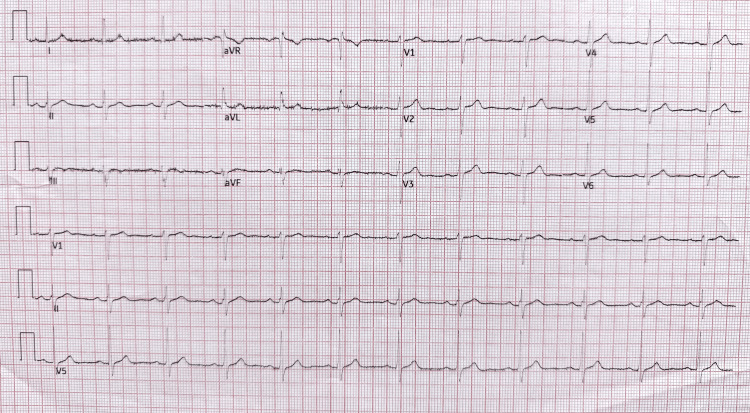
Repeat electrocardiogram, showing resolution of inferior lead (II, III, and aVF) ST-segment elevation. This figure also shows sinus rhythm at 71 beats per minute (bpm) and the resolution of the reciprocal changes in the lateral leads (I and aVL).

He was admitted to our cardiac monitoring unit for continuous cardiac monitoring, including telemetry, on suspicion of cardiac syncopal episodes. On his fourth day of admission, he experienced a five-minute episode of retrosternal chest discomfort while shaving, accompanied by waxing left-sided neck and shoulder pain. His initial ECG, after he reported experiencing chest discomfort, revealed ST-segment elevation in the inferior leads (Figure 2). This triggered the activation of our hospital's STEMI pathway, with prompt oral loading of aspirin and clopidogrel, and referral to the nearest PCI centre for immediate invasive coronary angiography and potential primary PCI. While awaiting transfer, his repeat ECG showed resolution of the inferior lead ST-segment elevation (Figure 3), and his high-sensitivity troponin T rose to 30 ng/L (reference: <14).

**Figure 4:**
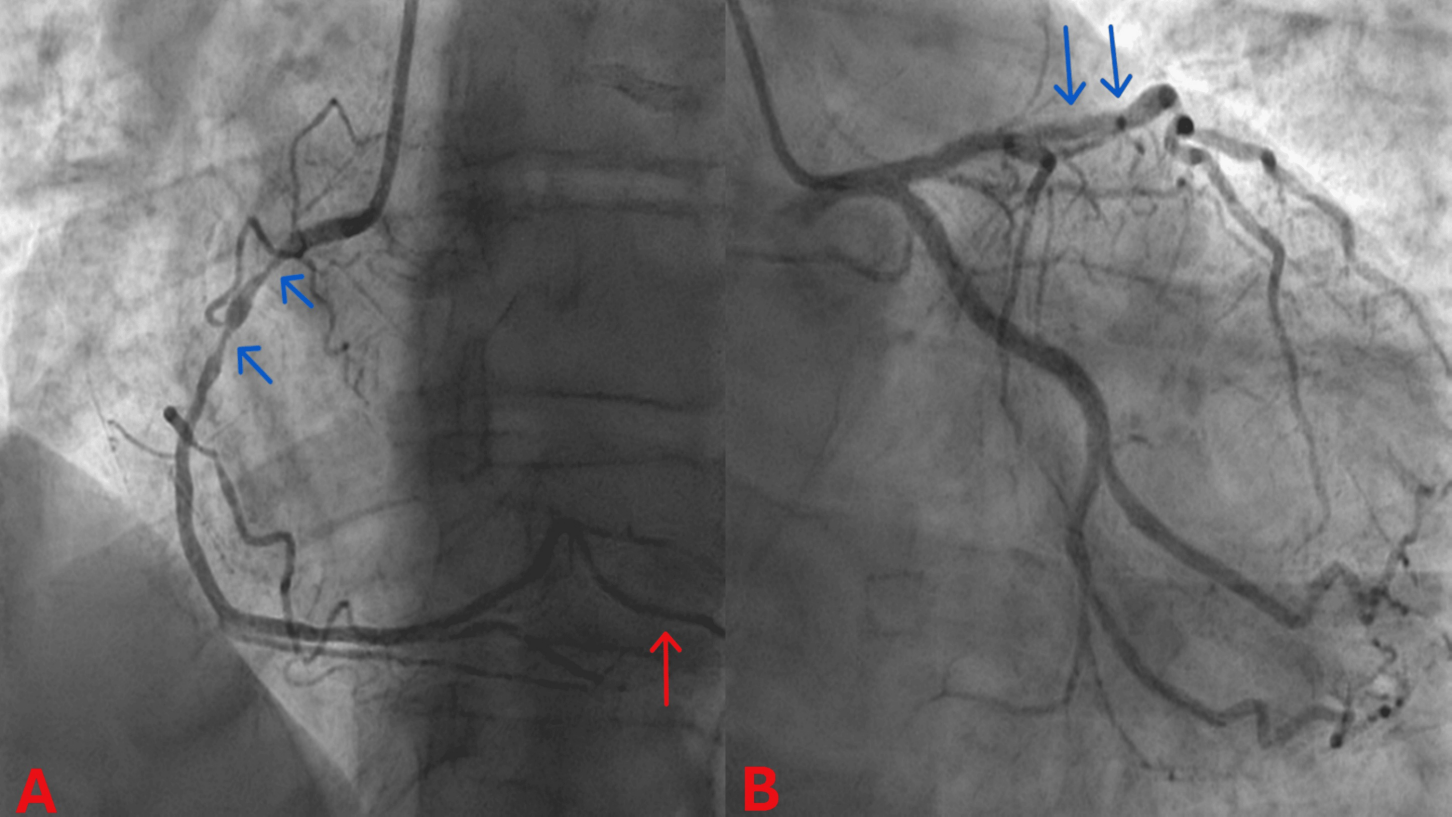
Coronary angiograms before percutaneous coronary intervention. (A) Left anterior oblique (LAO) angiogram of the right coronary artery (RCA). Blue arrows: severe proximal RCA stenosis; Red arrow: posterior descending artery (PDA) branch of the RCA (right-dominant coronary circulation). (B) Right anterior oblique (RAO) caudal angiogram of the left coronary artery (LCA). Blue arrows: moderate-to-severe mid-left anterior descending artery (LAD) stenosis.

**Figure 5:**
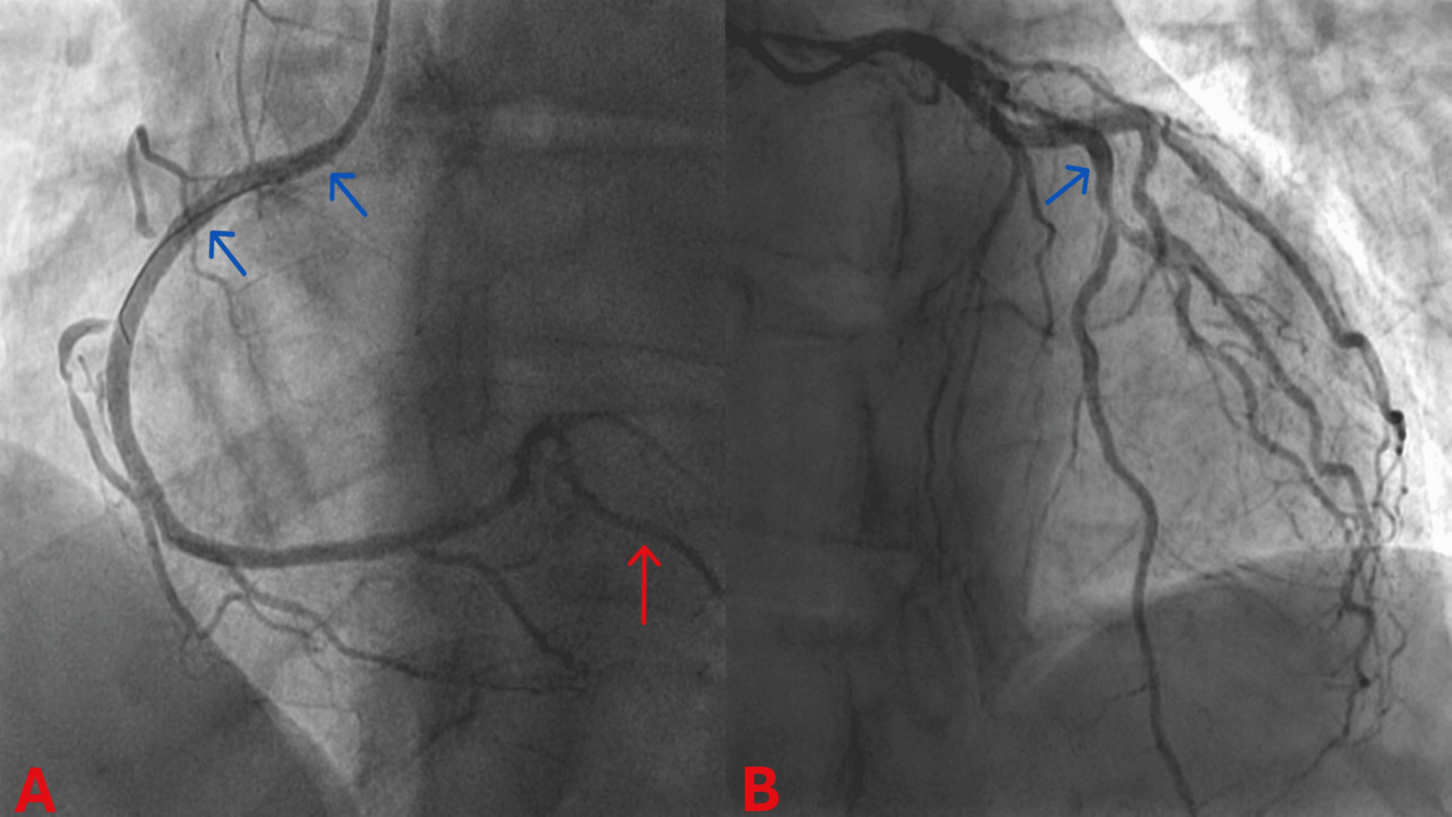
Coronary angiograms after percutaneous coronary intervention. (A) Left anterior oblique (LAO) cranial angiogram of the right coronary artery (RCA). Blue arrows: drug-eluting stent (DES) implantation site in the proximal RCA; Red arrow: posterior descending artery (PDA) branch of the RCA (right-dominant coronary circulation). (B) Right anterior oblique (RAO) cranial angiogram of the left coronary artery (LCA). Blue arrow: drug-eluting stent (DES) implantation site in the mid-left anterior descending artery (LAD).

On arrival at the PCI centre, he underwent an invasive coronary angiography, which revealed atherosclerotic CAD with severe proximal RCA stenosis and moderate-to-severe mid-LAD stenosis (Figure 4). He had PCI with DES implantation in his RCA, followed three days later by a staged intravascular ultrasound (IVUS) study and PCI with DES implantation in his LAD (Figure 5). He had a normal echocardiogram and was discharged the day after his staged PCI with no complications.

During a follow-up phone call six months after his discharge, he recounted how his left-sided neck and shoulder pain completely resolved immediately after his first PCI session, with no recurrence. He also confirmed that he had no further episodes of presyncope or syncope after his discharge. He reported having doubts about the initial diagnosis of musculoskeletal pain, given that his left-sided neck and shoulder pain felt different from his past experiences with musculoskeletal pain.

## Discussion

Our patient presented with episodes of syncope and chronic, ongoing pain on the left side of his neck and shoulder. During admission for cardiac monitoring, he experienced a transient episode of chest discomfort, which was associated with transient ST-segment elevation in his inferior ECG leads and a rise in serum troponin levels, leading to a diagnosis of NSTEMI. While STEMI is characterised by persistent ST-segment elevation (or ST-segment elevation equivalents) on ECG, the presence of transient ST-segment elevation in individuals with non-ST-elevation ACS (NSTEMI or UA) identifies a high-risk group in whom early (within 24 hours) invasive coronary angiography should be considered [2].

Our patient's invasive coronary angiogram revealed a two-vessel obstructive atherosclerotic CAD: severe proximal RCA stenosis and moderate-to-severe mid-LAD stenosis. His severe proximal RCA stenosis can explain most of his presenting clinical features. The RCA supplies blood to the right atrium and ventricle and, in most individuals, also significantly contributes to the blood supply of the left ventricle. The RCA supplies blood to the sinoatrial (SA) node in two-thirds of individuals through its SA nodal branch [11]. The AV node receives blood from the AV nodal branch of the dominant coronary artery, which is the RCA in most cases [11]. Coronary dominance is determined by the coronary artery that gives rise to the posterior descending artery (PDA) and the posterolateral branch (PLB) [11]. The RCA supplies the PDA and PLB in 70% of humans (right-dominance), while the LCx supplies them in 20% of cases (left-dominance) [11]. In the remaining 10% of cases (codominance), both the RCA and LCx give rise to the PDA and PLB [11]. The PDA supplies blood to the posteroinferior one-third of the interventricular septum and the inferior wall of the left ventricle [11]. Therefore, our patient's episodes of presyncope and syncope, which represent anginal equivalents, were most likely secondary to bradyarrhythmias induced by ischaemia of his SA and/or AV nodes. Although the sinus bradycardia and first-degree AV block he presented with at admission may have reflected a baseline increased vagal tone secondary to his athletic fitness, SA and AV node dysfunction due to ischaemia could have also contributed, respectively. To diagnose ischaemic SA and/or AV node dysfunction in clinical practice, ambulatory ECG monitoring and exercise testing may be necessary to correlate myocardial ischaemic symptoms with SA and/or AV node rhythm disturbances [12]. The transient chest discomfort, transient ST-segment elevation in inferior ECG leads, and serum troponin rise he experienced while in our cardiac monitoring unit indicate infarction of his left ventricular inferior wall, which is supplied by his dominant RCA (Figures 4A, 5A). Retrospectively, we also recognise that his chronic persistent left-sided neck and shoulder pain was an anginal equivalent secondary to his severe proximal RCA stenosis, as it resolved promptly following PCI with DES implantation in this artery.

Our patient’s symptom evolution from chronic unremitting pain to episodes of presyncope/syncope, culminating in an acute event (NSTEMI), suggests progressive, unstable atherosclerotic CAD. While persistent pain beyond 20 minutes is characteristic of ACS [2], this syndrome typically involves acute symptoms rather than the chronic pain our patient experienced. At admission, the initial ECG (Figure 1) did not show pathological Q waves, and the initial serum troponin was normal, suggesting that myocardial infarction may not have occurred during his symptomatic period before hospitalisation. However, a sensitive imaging test, such as cardiac magnetic resonance (CMR) [2], would have been required to rule out prior myocardial infarction definitively. Therefore, our patient's chronic unremitting pain may represent UA with a rather atypical, prolonged/chronic course.

From another perspective, the chronicity of our patient's left-sided neck and shoulder pain aligns more with CCS. Historically, pain in suspected CCS patients has been classified as typical, atypical, or non-anginal based on location, precipitating, and relieving factors [1]. Typical/classical angina refers to retrosternal chest pain/discomfort (or anginal equivalent) precipitated by stress (effort or emotion) and relieved by rest or nitroglycerin. Atypical angina meets two of these three characteristics of typical angina, while non-anginal pain has one or none. Studies show that while pain in the chest, jaw, neck, shoulder, arm, epigastrium, or back is the main symptom in up to 90% of suspected CCS cases, only 10%-25% of suspected cases report a history consistent with typical angina [5,6]. Lasting pain decreases the likelihood of CCS, and even among the many who present with less-than-typical angina, symptoms are usually episodic and transient due to reversible myocardial ischaemia [1].

Compared with obstructive atherosclerotic CAD, angina caused by epicardial vasospasm (epicardial vasospastic angina, or VSA) or coronary microvascular dysfunction (CMD) (microvascular angina, or MVA) is less likely to be classical [2]. Epicardial VSA predominantly occurs at rest and may show diurnal variation, being worse at night and in the early morning [13,14]. MVA may occur at rest or post-exercise, may not be relieved by nitroglycerin, and may have a prolonged duration [15,16]. The risk factors for obstructive atherosclerotic CAD, epicardial vasospasm, and CMD are similar, and these distinct pathologies can coexist [1]. Therefore, given our patient's multiple risk factors for atherosclerotic CAD (hypertension, dyslipidaemia, and smoking history), he may have also had coexisting epicardial vasospasm and/or CMD, contributing to the persistence of his pain. However, the complete resolution of his pain without recurrence following PCI makes additional mechanism(s) of myocardial ischaemia, other than atherosclerotic plaque obstruction, less likely. In clinical practice, the exclusion of epicardial vasospasm and CMD requires invasive coronary functional testing using acetylcholine and adenosine [1]. Another factor that may explain our patient's constant pain is abnormal cardiac nociception. It has been demonstrated that some angina patients may have an exaggerated perception of cardiac pain due to abnormalities in their neural pathways for cardiac nociception [17-19].

**Figure 6:**
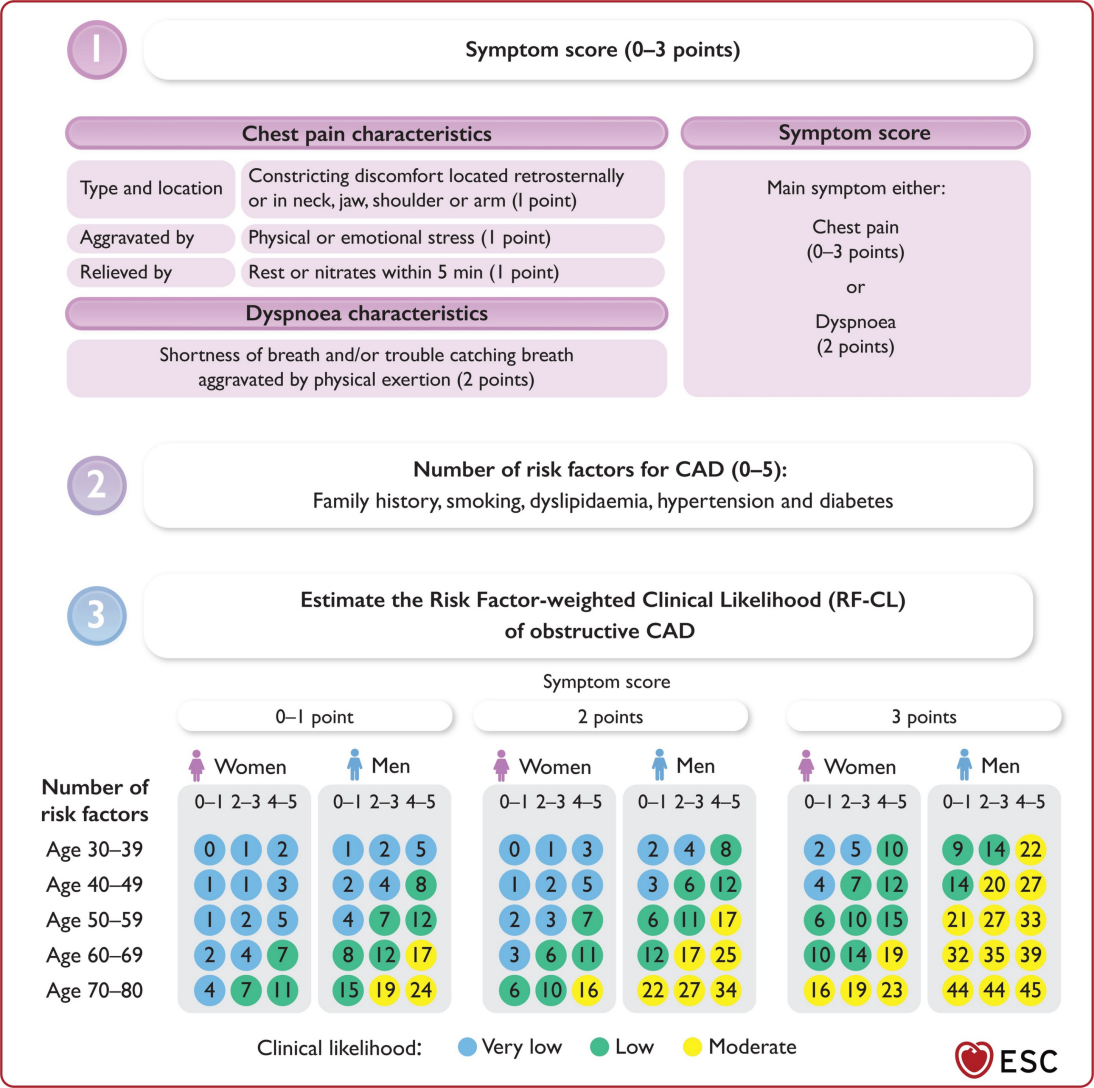
The RF-CL model for estimation of the clinical likelihood of obstructive atherosclerotic CAD. Image credit: Reproduced with permission from Vrints et al. [1]; copyright 2024, The European Society of Cardiology. CAD: coronary artery disease; RF-CL: risk factor-weighted clinical likelihood

The European Society of Cardiology (ESC) guidelines [1] recommend using the Risk Factor-Weighted Clinical Likelihood (RF-CL) model to estimate the pre-test clinical likelihood of obstructive atherosclerotic CAD in suspected cases of CCS (Figure 6). Estimates from this model are used to guide the choice of confirmatory test, with deferral of further testing, coronary computed tomography angiography (CCTA), functional imaging, and invasive coronary angiography recommended for patients with very low (≤5%), low or moderate (>5% to 50%), moderate or high (>15% to 85%), and very high (>85%) clinical likelihood estimates, respectively [1].

If the RF-CL model had been used to assess our patient's pre-test clinical likelihood of obstructive atherosclerotic CAD when he presented at his primary care with left-sided neck and shoulder pain, his age, sex, symptom score of 1, and three risk factors (ex-smoker, dyslipidaemia, and hypertension) would have estimated his pre-test clinical likelihood at 7% (Figure 6). This low clinical likelihood would have necessitated a CCTA, which would have revealed his obstructive CAD. However, the challenge with committing him to the CCS diagnostic pathway would have been the characteristics of his pain. He reported chronic, persistent pain and regularly engaged in intense physical activity, such as mountain biking, without any noticeable exacerbation of this pain - an unusual presentation for obstructive CAD. Therefore, it is clear that the unremitting nature of his anginal equivalent contributed to the missed diagnosis in primary care, where his history of preceding trauma led to a conclusion of musculoskeletal pain.

Besides the less-than-typical angina (or anginal equivalent) in many patients, the symptomatology of myocardial ischaemia may show considerable variation with age, sex, race, socioeconomic status, and geographical location [1]. Our patient's history showed that his principal myocardial ischaemic symptom varied over time: he had a remote history of dyspnoea on exertion, then developed chronic, persistent left-sided neck and shoulder pain unrelated to exertion, and finally, chest discomfort that heralded his NSTEMI. His variable symptomatology likely represents age-related variability.

Case reports of obstructive CAD presenting with persistent angina (or anginal equivalents) are rare in medical literature. We believe our patient's account of his pain being 'present all the time' is accurate, as he reported sleep disruptions. His engagement in physiotherapy and referral to a chiropractor further support the presence of persistent pain.

## Conclusions

Angina or its equivalents can, unusually, be chronic and persistent in obstructive atherosclerotic CAD, leading to missed or delayed diagnosis. The symptomatology of obstructive CAD may also vary over time. Our case highlights the importance of raising clinician awareness about atypical CAD presentations. Chest pain (or pain in other locations typical of myocardial ischaemia), dismissed as non-anginal due to atypical characteristics, such as persistence, should be dynamically reassessed for potential CAD, particularly when alternative treatments yield poor results. While our case may be a rare outlier, similar reports in the future could justify revising CCS diagnostic guidelines to better account for atypical presentations like our case.

## References

[1] Vrints C, Andreotti F, Koskinas KC (2024). 2024 ESC Guidelines for the management of chronic coronary syndromes. Eur Heart J.

[2] Byrne RA, Rossello X, Coughlan JJ (2023). 2023 ESC Guidelines for the management of acute coronary syndromes. Eur Heart J.

[3] GBD 2017 Causes of Death Collaborators (2018). Global, regional, and national age-sex-specific mortality for 282 causes of death in 195 countries and territories, 1980-2017: a systematic analysis for the Global Burden of Disease Study 2017. Lancet.

[4] Timmis A, Vardas P, Townsend N (2022). European Society of Cardiology: cardiovascular disease statistics 2021. Eur Heart J.

[5] Douglas PS, Hoffmann U, Patel MR (2015). Outcomes of anatomical versus functional testing for coronary artery disease. N Engl J Med.

[6] Douglas PS, Nanna MG, Kelsey MD (2023). Comparison of an initial risk-based testing strategy vs usual testing in stable symptomatic patients with suspected coronary artery disease: the PRECISE randomised clinical trial. JAMA Cardiol.

[7] Elliott MD, Heitner JF, Kim H (2019). Prevalence and prognosis of unrecognised myocardial infarction in asymptomatic patients with diabetes: a two-centre study with up to 5 years of follow-up. Diabetes Care.

[8] Yokota S, Ottervanger JP, Mouden M, de Boer MJ, Jager PL, Timmer JR (2018). Predictors of severe stenosis at invasive coronary angiography in patients with normal myocardial perfusion imaging. Neth Heart J.

[9] Fajadet J, Chieffo A (2012). Current management of left main coronary artery disease. Eur Heart J.

[10] Glazier JJ, Ramos-Parra B, Kaki A (2021). Therapeutic options for left main, left main equivalent, and three-vessel disease. Int J Angiol.

[11] Villa AD, Sammut E, Nair A, Rajani R, Bonamini R, Chiribiri A (2016). Coronary artery anomalies overview: the normal and the abnormal. World J Radiol.

[12] Sidhu S, Marine JE (2020). Evaluating and managing bradycardia. Trends Cardiovasc Med.

[13] Jenkins K, Pompei G, Ganzorig N, Brown S, Beltrame J, Kunadian V (2024). Vasospastic angina: a review on diagnostic approach and management. Ther Adv Cardiovasc Dis.

[14] Sinha A, Rahman H, Perera D (2022). Vasospastic angina: a contemporary review of its pathophysiology, diagnosis and management. Heart Int.

[15] Ong P, Camici PG, Beltrame JF (2018). International standardization of diagnostic criteria for microvascular angina. Int J Cardiol.

[16] Schindler TH, Dilsizian V (2020). Coronary microvascular dysfunction: clinical considerations and noninvasive diagnosis. JACC Cardiovasc Imaging.

[17] Agarwal M, Mehta PK, Bairey Merz CN (2010). Nonacute coronary syndrome anginal chest pain. Med Clin North Am.

[18] Cannon RO 3rd (2009). Microvascular angina and the continuing dilemma of chest pain with normal coronary angiograms. J Am Coll Cardiol.

[19] Garroni D, Fragasso G (2018). Heart or mind? Unexplained chest pain in patients with and without coronary disease. Heart and Mind.

